# Dehydroascorbic Acid Induces Cell Death in Sarcoma Stem Cells Under bFGF-Mediated Stemness-Supporting Conditions

**DOI:** 10.3390/antiox14111376

**Published:** 2025-11-19

**Authors:** Maja Ledinski, Katarina Caput Mihalić, Marijana Šimić Jovičić, Karla Ostojić, Zara Škibola, Robert Kolundžić, Inga Urlić

**Affiliations:** 1 Division of Molecular Biology, Department of Biology, Faculty of Science, University of Zagreb, 10 000 Zagreb, Croatia; maja.ledinski@biol.pmf.unizg.hr (M.L.); katarina.caput.mihalic@biol.pmf.unizg.hr (K.C.M.); karla.ostojic@biol.pmf.unizg.hr (K.O.); zara.skibola@mef.hr (Z.Š.); 2 Department of Transfusion and Regenerative Medicine, Sestre Milosrdnice University Hospital Center, 10 000 Zagreb, Croatia; 3 Department of Pediatric Orthopedics, Children’s Hospital Zagreb, 10 000 Zagreb, Croatia; marijana.simicjovicic@kdb.hr; 4 Department of Trauma Surgery, Sestre Milosrdnice University Hospital Center, 10 000 Zagreb, Croatia; rkolundzic@kbd.hr; 5 Department of Orthopaedics and Traumatology, University Hospital Dubrava, 10 040 Zagreb, Croatia

**Keywords:** ascorbate, sarcomas, cancer stem cells

## Abstract

The resilience of sarcomas, tumors characterized by resistance to therapy and high metastatic potential, is largely driven by the unique characteristics of a small population known as cancer stem cells (CSC). Although ascorbic acid (AA) and its oxidized form, dehydroascorbic acid (DHA), have shown potential for selectively targeting cancer cells, their effects on sarcoma CSCs remain insufficiently explored. Still, recent research indicates that AA can affect the specific characteristics of CSC and lead to their cytotoxicity. To investigate the sensitivity of sarcoma CSCs to ascorbate, CSCs were isolated from six sarcoma patient-derived samples using a sphere assay, and their stem identity was evaluated through gene expression profiling and dye-efflux assays. Cytotoxicity testing of AA and DHA showed that DHA has a selective cytotoxic effect on cancer stem cells. The presence of basic fibroblast growth factor (bFGF), which is commonly used to support the self-renewal of CSCs, had an influence on the cytotoxic effect of DHA. To evaluate the difference in the effect of AA and DHA, a seven-day treatment of CSCs with these forms of ascorbate was performed. The gene expression analysis revealed that DHA in the presence of bFGF had a stronger impact on response to oxidative stress and cellular metabolism. Also, investigation of somatic mutations of oncogenes and tumor suppressors revealed that in liposarcoma and rhabdomyosarcoma, there are mutations that induce proliferative signals. These proliferative signals, joined with bFGF in the presence of DHA, do not lead to proliferation but instead cause cell death.

## 1. Introduction

Sarcomas are rare but diverse and highly aggressive malignant neoplasms that arise from mesenchymal cells, affecting bone and connective tissues. Although rare in adulthood, they present about 15% pediatric tumors. Despite different therapy models, sarcomas are very deadly, as one-third of the affected patients die of sarcoma [1]. Historically, they have been classified into two major groups: bone sarcomas and soft tissue sarcomas. However, there are numerous histological subtypes, and they can occur in nearly any anatomical location in the body. This results in a wide range of possible combinations between histological subtypes and tumor locations, which complicates both diagnosis and treatment approaches [2]. Additionally, sarcomas show high resistance to both chemotherapy and irradiation therapy, which makes their treatment very difficult. This high resistance to therapeutic approaches, invasiveness, and heterogeneity observed in sarcomas can be explained by the cancer stem cell (CSC) theory [3,4]. This theory states that a single initiating cell, possessing distinct self-renewal capabilities and specific mutations, can give rise to the entire tumor population [5,6]. Through division, it generates diverse differentiated progeny and other cancer stem cells. Initially described in hematopoietic cancers, they have since been found in many different types of cancers, including sarcomas [3]. Apart from expressing stem cell markers (OCT4, SOX2, NANOG), they are also characterized by surface markers (CD44, CD133). However, there is still no universal CSC marker, which makes it harder to detect them [7,8]. Additionally, CSC exhibit a predominantly quiescent metabolism, which can dynamically shift between glycolysis—and oxidative phosphorylation—dependent metabolic phenotypes [8,9,10,11]. This metabolic adaptability poses significant challenges for the development of therapeutic strategies aimed at selectively targeting their energetic pathways.

As a well-known antioxidant, vitamin C plays diverse roles since it is an essential cofactor in numerous enzymatic reactions and serves as a key participant in various biosynthetic pathways. It enters the cells in two different forms—as ascorbic acid (AA) through sodium-dependent channels SVCT-1 encoded by gene *SLC23A1* and SVCT-2 encoded by *SLC23A2*. In the extracellular space, ascorbate is oxidized into DHA by reactive oxygen species (ROS). Dehydroascorbic acid (DHA), the fully oxidized form of ascorbate, can be present in a shape that is structurally similar to glucose, which enables it to enter the cells via GLUT channels, transmembrane proteins that facilitate passive import of glucose [12]. Since tumors react to hypoxia and poor nutrient conditions by upregulating expression of glucose transporters [6], they enable greater uptake of ascorbate through GLUTs, predominantly through GLUT1 and GLUT3 [13]. Upon entering the cells, DHA is converted to ascorbate at the expense of the reducing agents in the cell (glutathione and NADPH), resulting in an increase in intracellular ROS [13]. Generally, cells have higher concentrations of ascorbate (1–10 mM) than the human plasma (~50 μM). However, interesting examples are hematopoietic stem cells, which have 18× higher content of ascorbate than their differentiated progeny [14].

Although the use of ascorbate in cancer therapy remains a topic of considerable debate, numerous studies have investigated its potential anti-tumor effects, which are mostly attributed to its pro-oxidative activity within cancer cells and the resulting selective pressure in malignant tissues [13,15]. Since cancer cells often overexpress GLUT transporters to facilitate increased glucose uptake due to their reliance on glycolysis for energy production, DHA can readily enter these cells via the relatively nonspecific GLUT transporters by mimicking glucose [16]. However, DHA quickly converts to ascorbyl radical and AA at the expense of NADPH and glutathione, which leads to exhaustion of the antioxidative defense mechanisms of cancer cells [12,13]. Further generation of ROS leads to damaging effects on DNA, proteins, and lipids of cancer cells. As cancer cells have increased levels of Fe^2+^, they can react with H_2_O_2_ to generate hydroxyl radical, another powerful ROS [15]. Although induction of oxidative stress is considered a main mechanism of action by which ascorbate can selectively affect cancer cells, some studies suggest a few additional mechanisms. Vitamin C is essential for collagen stability, which helps prevent metastatic invasion by maintaining extracellular matrix stability [14]. Vitamin C also downregulates HIF-1 transcription factor, which is key for tumor proliferation in hypoxic environments. Further, from the same family of enzymes, there are TET enzymes, which regulate the methylation of DNA. In leukemic cells, it was found that vitamin C acts as a cofactor for TET enzymes and its presence can, as a consequence, restore the expression of tumor suppressor genes [12,13]. Metabolomic studies have been conducted in breast [17] and colorectal [18] carcinoma cells following administration of high-dose vitamin C. These studies consistently demonstrated an accumulation of metabolites upstream of glyceraldehyde 3-phosphate dehydrogenase (GAPDH) within the glycolytic pathway. These findings confirm earlier observations by Yun et al. in colorectal cancer cells, confirming that glycolysis is disrupted upon treatment with pharmacological concentrations of vitamin C [18].

In order to study CSC behavior in vitro, establishing appropriate culture conditions is essential, as this enables accurate investigation of their biology and the identification of potential therapeutic vulnerabilities. That is why, in culturing CSC, bFGF is a crucial factor for maintenance of their stemness characteristics as well as induction of their proliferative capacity [19,20]. Briefly, there are four types of FGF receptors (FGFR 1–4) and 23 FGFs, and it is considered that 4% sarcoma patients have mutations in FGFRs [21]. Through FGFRs, signals can activate RAS-MAPK and PI3K-PKB pathways. bFGF acts as a ligand for FGFR1–4 and has important roles in cell proliferation, migration, and differentiation [22]. However, in sarcomas, FGFRs can be overexpressed or have activating mutations, which can affect the binding of FGFs and cause different downstream effects. For example, in rhabdomyosarcoma, activating mutations were found in the tyrosine kinase domain of FGFR4, which enable easier phosphorylation and activation of downstream pathways. Additionally, a study on rhabdomyosarcoma found that it has a population of FGF3-positive cells with stem cell characteristics. In liposarcoma, activating mutations were found in FGFR1 and FGFR3, as well as overexpression of FGFR1 and FGFR4, both of which are related to poor prognosis in patients. FGFR inhibitors showed promising results in preclinical studies as well as clinical trials [21]. Therefore, when investigating CSC, it is important to consider the role of bFGF in response to therapy.

Although AA appears to be a promising agent for targeting CSCs, either as a monotherapy treatment or in combination with conventional chemotherapeutics, the effects of AA on CSCs in sarcomas remain unclear. To address this question, we isolated CSC populations from six distinct sarcoma samples. Following characterization of these CSCs, we investigated the cytotoxicity of both AA and its oxidized DHA and subsequently, using gene expression analysis, aimed to reveal which processes in the cells are affected by the treatment.

## 2. Materials and Methods

### 2.1. Cell Culture

Human embryonic kidney HEK 293 and human osteosarcoma U2OS cell lines were kindly provided by Vjekoslav Tomaić (Ruđer Bošković Institute, Zagreb, Croatia). HEK 293 and U2OS cell lines were cultured in DMEM High glucose (4.5 g/L) supplemented with 10% fetal bovine serum (FBS) and 1% penicillin/streptomycin. Cells were cultured at 37 °C in the presence of 5% CO_2_. Human mesenchymal stem cells (hMSC) were obtained from Cytion (Eppelheim, Germany) and cultured in low-glucose (1 g/L) DMEM supplemented with 10% FBS, 1% penicillin/streptomycin, and 0.01% bFGF.

#### 2.1.1. Establishing Parental Sarcoma Cell Culture

Sarcoma samples were isolated from patients’ tumor tissue during diagnostic biopsies (with approval of the Ethics Committee of the School of Medicine, University of Zagreb, nr. 380-59-10106-23-111/91) and stored in a tube with growth medium (Coon’s modification Ham’s F12 medium, 10% FBS, 1% pen/strep). The sample was sent to the cell culture laboratory, where, under sterile conditions, it was washed with phosphate-buffered saline (PBS) and cut into small pieces (0.5–1 mm^2^). The pieces were then subjected to digestion in a medium with collagenase type II (Coon’s modified Ham’s F12 medium, 20% FBS, 1% pen/strep, 3.75 mg/mL collagenase type II) for two hours at 37 °C, 5% CO_2_. After the digestion, the suspension was centrifuged at 300× *g*, 10 min, the supernatant was aspirated, and the precipitate was resuspended with growth medium and seeded into the adherent cell culture plate (T-150 flask) at 37 °C, 5% CO_2_. The next day, cell attachment was checked by microscopy. The growth medium was changed twice a week. The cells were passaged at 70% confluency. In this study, passages 1–5 were used.

#### 2.1.2. Sarcoma Stem Cell Isolation

CSCs were isolated by the sarcosphere assay. Parental sarcoma cells were detached by trypsinization, centrifuged at 350× *g* for 5 min, and resuspended in 2× medium for the sarcosphere assay. The medium was prepared by dissolving Coon’s modification of Ham’s F12 medium powder in half as much water, then sterile-filtered and supplemented with 2% ITS. After cell counting, the cell suspension (4 × 10^4^ cells/well) was mixed with 2% methylcellulose and seeded into ultra-low attachment six-well plates. Cells were supplemented with 0.01% EGF and 0.01% bFGF on the day of seeding and consecutively every three days. Sarcosphere growth was observed by a microscope. After 2–4 weeks, sarcospheres were isolated using a 40 µm nylon cell strainer and seeded into adherent cell culture plates in growth medium with 0.01% bFGF. Upon adherence, the first generation of CSCs was thereby isolated and propagated. To generate the second generation of CSCs, the entire procedure was repeated using the first-generation sarcoma CSCs. Upon isolation of the second generation of SSCs, they were propagated and used for experiments in passages 1–8.

### 2.2. Sarcoma Stem Cell Characterization

#### 2.2.1. Hoechst 33342 Dye Efflux Assay

CSCs and U2OS as control cells were seeded in a black 96-well plate (6 × 10^4^ cells/well) and allowed to adhere overnight at 37 °C, 5% CO_2_. The next day, cells were treated with 5 µg/mL Hoechst 33342 in 100 µL of appropriate growth medium and incubated for two hours at 37 °C, 5% CO_2_. Afterwards, the treatment was aspirated, and cells were washed with cold PBS in the dark. The fluorescence was measured by the GloMax microplate reader (Promega, Madison, WI, USA) at excitation 360 nm, emission 450 nm.

#### 2.2.2. qPCR Stemness Marker Analysis

RNA was isolated from CSCs (Zymo Research, Irvine, CA, USA), and its concentration and quality were measured by NanoVue Plus Spectrophotometer (Biochrom, Cambridge, UK). Afterwards, RNA was DNase I-treated (DNase I, RNase-free, Thermo Fisher Scientific, Waltham, MA, USA) and reverse transcribed (iScript cDNA Synthesis kit, Bio-Rad, Hercules, CA, USA). The prepared cDNA was used for qPCR experiments. qPCR was performed using SsoAdvanced Universal SYBR Green Supermix (Bio-Rad) on a CFX Opus 96 Real-Time PCR system under the following thermal cycling protocol: 30 s at 95 °C, 40 cycles of 15 s at 95 °C and 30 s at 60 °C. Primers used are listed in Table 1 [23]. qPCR was performed with two controls—no-template control (Milli-Q water instead of template) and no-RT control (to evaluate residual genomic DNA in isolated RNA). Relative quantification of the gene expression was performed using the 2^−∆∆Ct^ method.

### 2.3. MTT Viability Assay

Cells were seeded in a 96-well plate (1 × 10^4^ cells/well) and allowed to adhere overnight at 37 °C, 5% CO_2_. Treatments with AA and DHA were prepared in cell culture medium. Cells were treated in triplicate for 72 h. Following treatment, the medium was aspirated, and the cells were washed three times with 200 μL of PBS to eliminate potential interference of ascorbic acid with the MTT reagent. Cells were incubated for 4 h with MTT solution in cell culture medium at a concentration of 0.5 mg/mL, and upon incubation, 170 μL DMSO was added directly to the MTT solution on the cells to dissolve the formazan crystals. Absorbance was measured at 570 nm using a GloMax microplate reader (Promega, Madison, WI, USA), and cell viability was calculated as a percentage of the untreated control after subtracting the blank absorbance from the absorbance of all samples.

### 2.4. Assessment of Changes in Metabolism, Response to Oxidative Stress, and Differentiation

Cells were seeded into tissue culture-treated six-well plates in DMEM/F12, 10% FBS, 1% pen/strep, and 0.01% bFGF and left to adhere overnight at 37 °C, 5% CO_2_. Treatments with 1 mM AA and 1 mM DHA in culture medium were prepared in the dark. Growth medium was aspirated, and cells were treated for 7 days with treatment media changes every other day. On the last day of treatment, the media was aspirated, and the cells were detached by trypsinization. DNA, RNA, and proteins were isolated using the Zymo Research kit.

#### 2.4.1. qPCR

Gene expression analysis was performed as described in Section 2.2.2. with primer sequences listed in Table 2 [23]. Primer sequences for the *GAPDH* gene were obtained from reference [24], and the GPX1 gene was obtained from reference [25].

#### 2.4.2. Western Blot

Bicinchoninic Acid (BCA) Protein Assay Kit (Santa Cruz Biotechnology, Dallas, TX, USA) was used to determine the concentration of isolated proteins. Absorbance was measured at 560 nm using the Glomax microplate reader (Promega). Samples were prepared for SDS-PAGE using 4× Laemmli Sample Buffer (Bio-Rad) and heated at 95 °C for 5 min. Gels for electrophoresis were prepared using TGX Stain-Free FastCast Acrylamide Solutions (Bio-Rad). Upon polymerization, gels were placed in the Mini-Protean TETRA System (Bio-Rad). After loading the samples (5 μg per sample), electrophoresis was run at 180 V for 100 min. Proteins were transferred to a nitrocellulose membrane (Amersham^TM^ Protran^TM^, GE Healthcare Life Science, San Francisco, CA, USA) using Mini Trans-Blot Electrophoretic Transfer Cell (Bio-Rad) at 350 mA for 1 h. Membrane was blocked in 5% non-fat milk TBST pH 7.4 for 1 h and incubated with primary antibody GAPDH (D16H11) XP^®^ Rabbit mAb (Cell Signaling Technology, Danvers, MA, USA) diluted in 5% non-fat milk in 1× TBST in ratio 1:5000 overnight at 4 °C. Membrane was washed in TBST and incubated with secondary antibody Anti-Rabbit IgG HRP-linked Antibody (Cell Signaling Technology) diluted in 0.5% non-fat milk in 1× TBST in ratio 1:2500. Membrane was incubated with Clarity Western ECL Substrate (Bio-Rad) and visualized using ChemiDoc Imaging System (Bio-Rad). In the absence of a loading control, to account for the unequal loading of protein samples, the densitometric analysis was performed, and GAPDH protein band intensities were quantified and normalized to the total protein signal for each corresponding sample. The resulting normalized values were used for data presentation in the graphs.

### 2.5. Somatic Mutation Gene Panels

Analysis of the somatic mutations of the selected sarcoma samples was performed using the qBiomarker Somatic Mutation PCR Array Human Soft Tissue Tumors array. This array tests for the 83 specific mutations often found in soft tissue sarcomas. To perform the test, DNA isolated from samples using the DNA isolation kit (Qiagen, Hilden, Germany) was measured to determine concentration and purity ratios. After that, DNA was mixed with the qBiomarker Probe Mastermix and loaded onto the qBiomarker Somatic Mutation PCR array plate. The plate was covered with optical, thin-wall, eight-cap strips and shortly centrifuged to remove the bubbles. The cycling program was set up on a CFX Opus 96 Real-Time PCR system as follows: 10 min 95 °C, 40 cycles of 15 s 95 °C and 60 s 60 °C. The obtained Cq values were evaluated according to the manufacturer’s instructions to determine the presence of mutations in the samples.

## 3. Results

### 3.1. Sarcoma Cancer Stem Cells Isolation and Characterization

**Figure 1:**
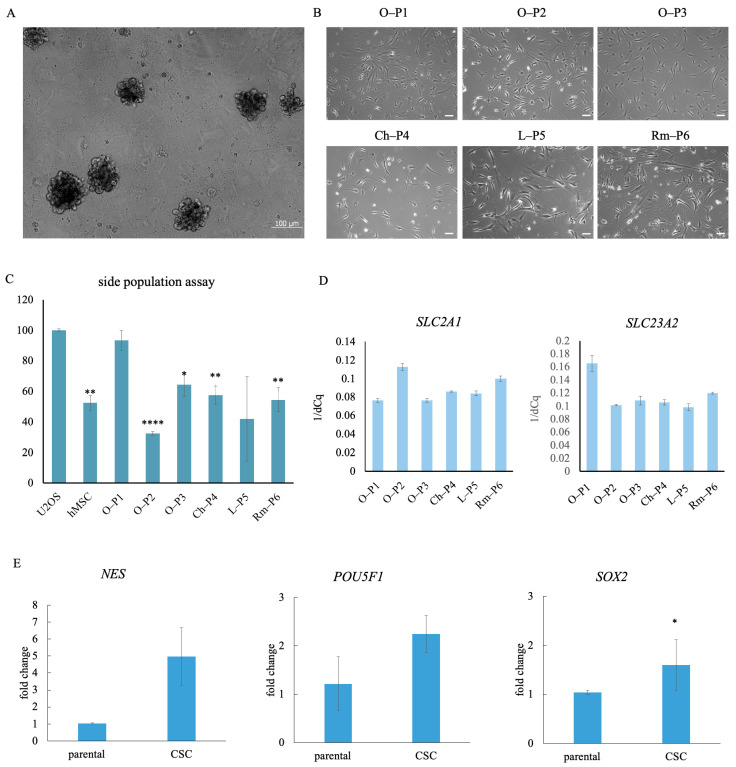
Characterization of cancer stem cells isolated from three osteosarcoma samples, O-P1, O-P2, and O-P3, chondrosarcoma sample, Ch-P4, liposarcoma sample, L-P5, and rhabdomyosarcoma sample, Rm-P6. (**A**) Sarcoma stem cells grow as sarcospheres. (**B**) CSCs isolated from six different sarcoma samples exhibit diverse morphologies. (**C**) Side population assay for control cells (U2OS and hMSC) and sarcoma samples, *n* = 3, statistically significant difference compared to the negative control U2OS is indicated by * (* *p* < 0.5, ** *p* ≤ 0.01, **** *p* ≤ 0.0001). (**D**) Relative gene expression analysis of *SLC2A1*, which encodes GLUT1 and *SLC23A2*, which encodes SVCT-2, *n* = 3. (**E**) Relative gene expression analysis of stemness markers in parental sarcoma cell lines and CSCs isolated after culturing in two generations of sarcospheres. The data are presented as the mean ± standard deviation; *n* = 5 for NES and *n* = 3 for *POUF1* and *SOX2.* A statistically significant difference in gene expression compared to the parental cells is indicated by * (*p* < 0.5).

When cells isolated from sarcoma samples were cultured under non-adherent and nutrient-deprived conditions, in all six of the parental sarcoma cultures, only a small subset of cells was able to grow and create spheres, a hallmark of stem-like properties (Figure 1A). During the sphere assay, while some cells proliferated and created spheres, others underwent cell death. Upon transfer to adherent culture conditions, the spheres attached to the cell culture flask surface, which allowed cells to migrate outward and spread, ultimately growing as a monolayer (Figure 1B).

Side population assay was conducted to evaluate the ability of CSCs to efflux the Hoechst 33342 dye. As shown in Figure 1C, hMSC, the positive control, exhibited approximately half the fluorescence intensity of the negative control, the U2OS cell line. CSCs derived from various sarcoma samples generally demonstrated fluorescence levels comparable to the hMSC positive control, indicating a similar dye-efflux phenotype, except for the osteosarcoma CSC sample O-P1, which displayed a different fluorescence profile.

To evaluate the expression of RNA for transporters that are critical for ascorbate uptake, *SLC2A1* and *SLC23A2* were analyzed by qPCR. Assessing these transport channels was important to enable potential correlation of their expression levels with the sensitivity of sarcoma samples to ascorbate treatment. The expression of the *SLC2A1* gene, which encodes the GLUT1 protein responsible for glucose transport and through which DHA efficiently enters cells, was found to be evenly expressed in O-P1, O-P3, Ch-P4, and L-P5 samples, while its expression was elevated in O-P2 and Rm-P6 (Figure 1D). On the other hand, the *SLC23A2* gene, which encodes SVCT-2, the transporter for AA, was uniformly expressed in the CSC samples O-P2, O-P3, Ch-P4, L-P5, and Rm-P6, whereas its expression was higher in the CSC sample O-P1 (Figure 1D).

Analysis of stemness markers showed genes *NES*, *POUF1,* and *SOX2* all have a greater expression in CSC than in the parental cell lines of sarcomas, with *SOX2* being significantly more expressed in the CSC cell line. While *POUF1* and *SOX2* show increased expression (*SOX2* with statistical significance), *NES* shows five times higher expression in CSC (Figure 1E).

### 3.2. Differential Viability Responses to Ascorbate Derivatives in Normal Cells, Osteosarcoma Cell Line, and Sarcoma CSCs

To assess the effect of ascorbate on non-tumor cells, the HEK293 and hMSC cell lines were used as controls, whereas the U2OS osteosarcoma cell line served as a control for differentiated tumor cells. At concentrations up to 1 mM, AA has almost no effect on the viability of HEK293 cells, but between 1 and 5 mM, there is a sharp decrease in viability, and at 10 mM AA, no viable cells remain. Treatment of HEK293 cells with DHA results in a very small effect on viability at concentrations between 0.5 mM and 10 mM. When treating U2OS cells, it is observed that AA causes a slight increase in viability, but at 1 mM, viability drops to about 40%, while at 5 and 10 mM, no viable cells remain. On the other hand, treatment with DHA results in only a very mild effect on viability, noticeable only at 5 mM, with 50% of cells remaining viable at 10 mM.

**Figure 2:**
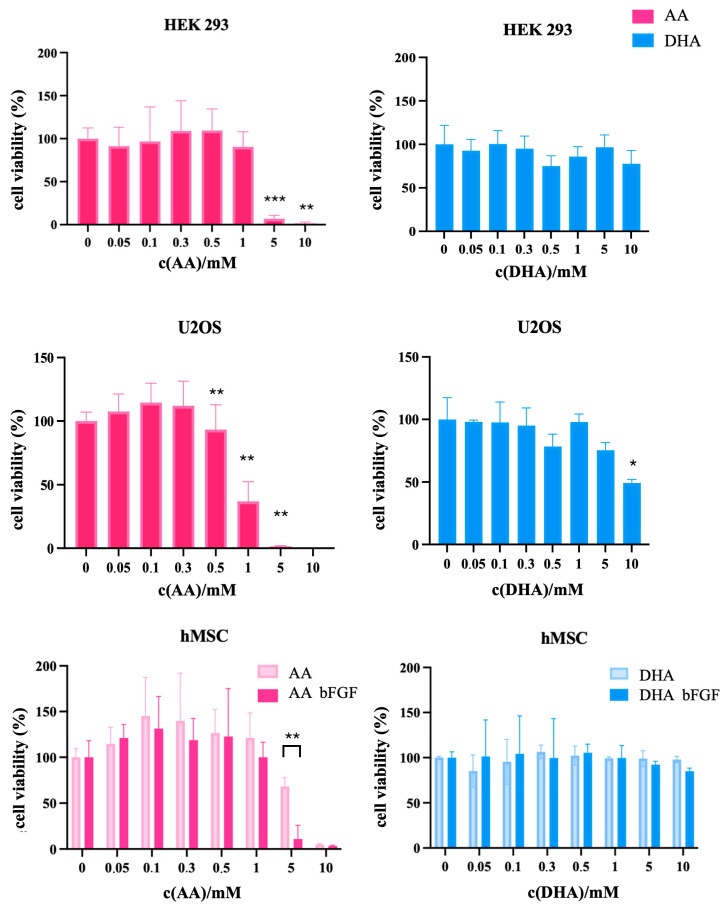
MTT viability assay in control cell lines HEK293, U2OS, and hMSC. HEK293 and U2OS were treated with both ascorbic acid (AA) and dehydroascorbic acid (DHA). When treating hMSCs, AA and DHA were applied in the presence and absence of bFGF to account for its possible influence on cell viability. Data are expressed as percent of negative control, mean ± sd, *n* = 3. Welch *t*-tests were performed. Statistical significance: * *p* ≤ 0.05, ** *p* ≤ 0.01, *** *p* ≤ 0.001.

When treating hMSC, AA at concentrations up to 1 mM causes a slight increase in viability, both in culture media with bFGF and without it. At 5 mM, there is a more pronounced drop in viability in the medium containing bFGF, while in the medium without bFGF, viability decreases gradually between 1 and 10 mM. In contrast, treatment with DHA shows no impact on viability at any concentration or in either culture condition, which is a desirable outcome for control cells (Figure 2).

**Figure 3:**
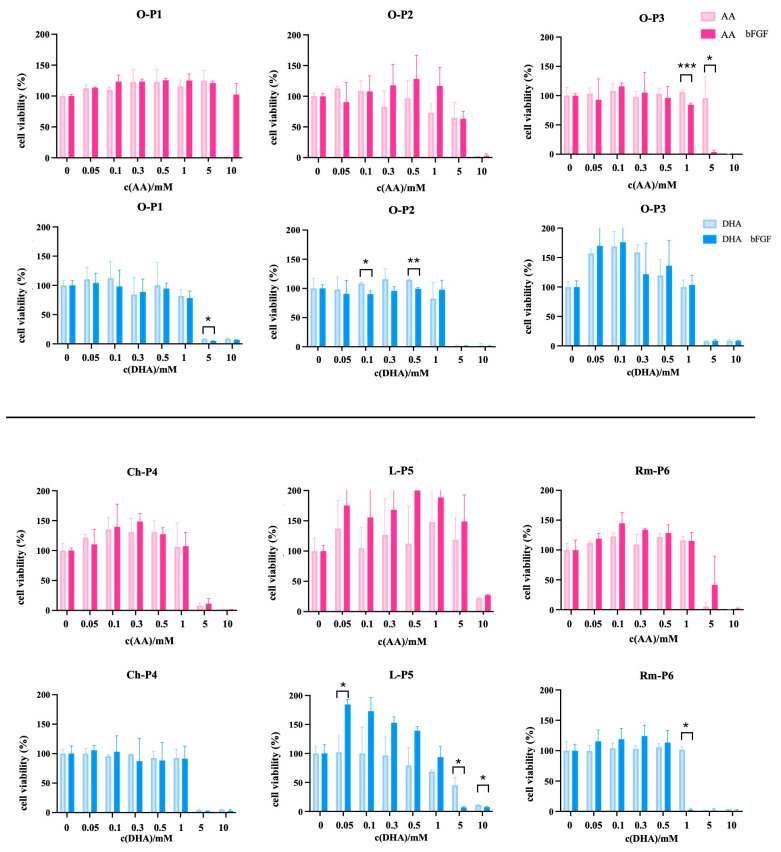
MTT viability assay in CSC isolated from different sarcoma samples, three osteosarcoma samples, O-P1, O-P2, and O-P3, chondrosarcoma sample, Ch-P4, liposarcoma sample, L-P5, and rhabdomyosarcoma sample, Rm-P6. Treatments with AA and DHA were applied in both the presence and absence of bFGF to account for its possible influence on cell viability. Data are expressed as percent of negative control, mean ± sd, *n* = 3. Welch *t*-tests were performed. Statistical significance: * *p* ≤ 0.05, ** *p* ≤ 0.01, *** *p* ≤ 0.001.

After testing control cell lines, CSC from sarcoma samples were treated for 72 h with AA or DHA in conditions with/without bFGF to evaluate whether the absence of bFGF affects the cytotoxicity of these forms of ascorbate (Figure 3). When treating O-P1 with AA in the presence of bFGF, there is no major effect on the change in viability. However, at 10 mM without bFGF in culturing media, there are no viable cells. The same cells react differently to treatment with DHA: at concentrations 1–10 mM, regardless of bFGF, there is a decrease in viability, abruptly from 1 to 5 mM. When applying AA to O-P2, there is a notable difference in treatments with/without bFGF in the medium. The cells with bFGF in the culturing medium show a slight increase in viability, followed by a gradual decrease from 1 to 10 mM. However, when treating the same cells with DHA, they show a rapid decrease in viability between 1 and 5 mM. AA exhibits an effect on O-P3 at 5 mM with bFGF in culturing medium, while at the same concentration, cells cultured in medium without bFGF show 100% viability. When treating O-P3 CSCs with DHA, they show an increase in viability, followed by a sudden drop at 5 mM, regardless of bFGF (Figure 3).

Regarding the CSCs from chondrosarcomas and soft tissue sarcomas, chondrosarcoma CSC, Ch-P4, react to treatments with AA and DHA with an abrupt decrease in viability between 5 and 10 mM regardless of the absence/presence of bFGF in the culturing medium. However, treatment with AA stimulates the growth of CSC up to 1 mM. When treating L-P5 with AA and DHA, there is a tangible difference in treatments regarding the absence of bFGF, since treatments with AA or DHA up to 1 mM greatly increase the viability of CSCs if there is bFGF in the culturing medium. AA achieves a decrease in viability up to ~20% at 10 mM regardless of bFGF in the medium. However, when treating with DHA, there is a difference, as there is a gradual decrease in viability from 0.5 mM when there is no bFGF in the culturing medium. When applying DHA with bFGF, there is a rapid decrease in viability between 1 and 5 mM. Finally, when treating Rm-P6 with AA, there is an increase in viability up to 1 mM and a decrease in viability between 1 and 5 mM, which is more gradual if there is bFGF present in the culturing medium. When treating the same cells with DHA, there is no increase in viability. However, 1 mM DHA without bFGF in the medium preserves 100% viability, while bFGF treatment causes a full decrease in viability (Figure 3).

### 3.3. Modulation of Gene Expression Related to Oxidative Stress, Metabolism, and Differentiation in Sarcoma CSCs by Ascorbate Treatments in the Presence of bFGF

**Figure 4:**
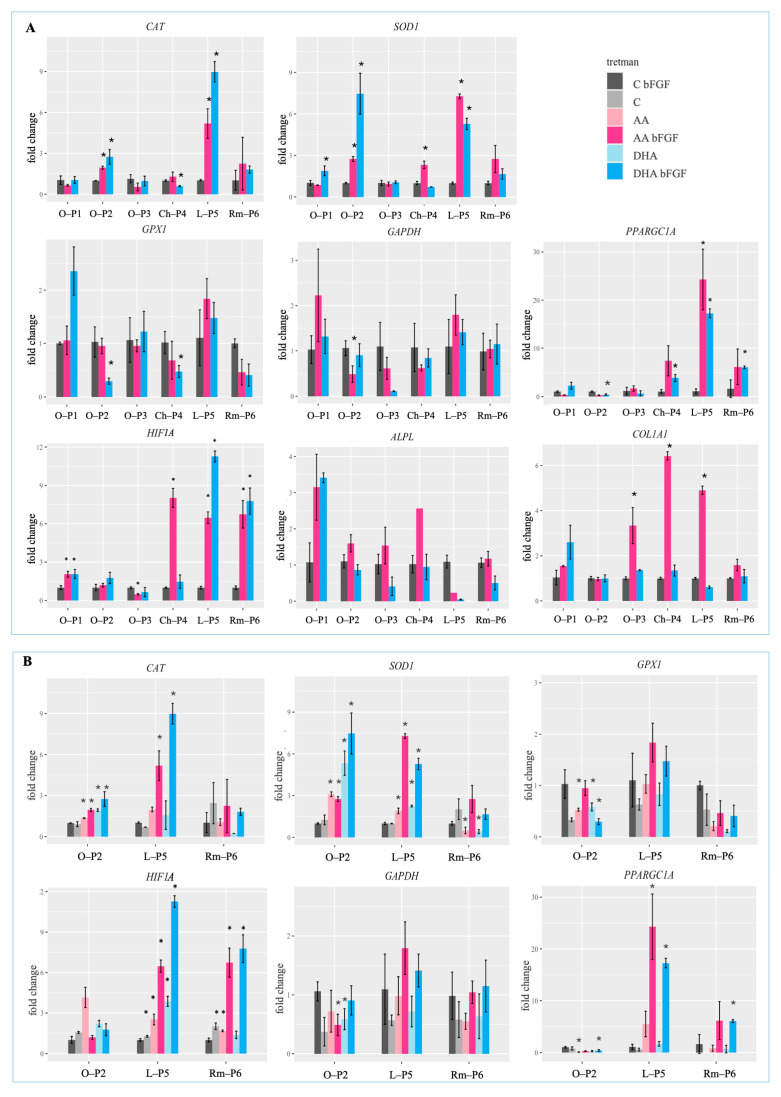
Relative gene expression analysis of genes associated with oxidative stress, metabolism, and differentiation. (**A**) Analysis of gene expression in cancer stem cells isolated from patients’ osteosarcoma samples, O-P1, O-P2, and O-P3, as well as chondrosarcoma, Ch-P4, liposarcoma, L-P5, and rhabdomyosarcoma, Rm-P6. (**B**) Additional gene expression analysis of cancer stem cells isolated from three selected sarcoma samples, O-P2, L-P5, and Rm-P6, including treatments without bFGF. C bFGF—CSC cultured in medium with bFGF; AA—CSC cultured in medium with 1 mM ascorbic acid and 0.01% bFGF; DHA—CSC cultured in medium with 1 mM dehydroascorbic acid and 0.01% bFGF. Data are expressed as percent of negative control, mean ± sd, *n* = 3. Welch *t*-tests were performed. A statistically significant difference compared to the C bFGF group is indicated by * (*p* < 0.5).

Since CSCs should be cultured in medium with bFGF, in the experiments, this condition is considered a negative control. After a 7-day treatment with AA and DHA, RNA was isolated, and expression of markers of oxidative stress, metabolism, and differentiation was evaluated. Regarding the expression of *CAT*, treatments with AA bFGF and DHA bFGF induce its expression in O-P2 and L-P5, with DHA bFGF having a stronger effect compared to AA bFGF (Figure 4A). Expression of *SOD1* is upregulated both in osteosarcomas O-P1 and O-P2, as well as soft tissue and chondrosarcoma samples. When treating osteosarcomas, treatment with DHA bFGF in samples O-P1 and O-P2 exerts a stronger influence on *SOD1* expression compared to treatments with AA bFGF, while in Ch-P4, L-P5, and Rm-P6, the effect was the opposite. When investigating the expression of *GPX1*, the greatest increase in expression is observed in the O-P1 sample with DHA bFGF treatment and in the L-P5 sample with both AA bFGF and DHA bFGF treatments. However, in the samples O-P2, H-P4, and Rm-P6, both treatments decrease *GPX1* gene expression (Figure 4A). When observing *HIF1A* expression, its expression slightly increased in O-P1, but in Ch-P4, it is increased by AA bFGF, and in L-P5 and Rm-P6, both AA bFGF and DHA bFGF increase its expression significantly (Figure 4A).

Regarding the expression of *GAPDH*, in the case of O-P1 and L-P5 treatment with AA, bFGF shows a slight increase in the expression of *GAPDH,* while the same effect is not observed for DHA bFGF. However, in samples O-P2, O-P3, and Ch-P4, there is a slight decrease in expression of *GAPDH* induced by both treatments (Figure 4A). When investigating the expression of *PPARGC1A*, in the samples Ch-P4, L-P5, and Rm-P6, treatments with AA bFGF and DHA bFGF lead to an increase in its expression, while this effect is not observed in osteosarcomas.

Regarding expression of differentiation markers, treatments AA bFGF and DHA bFGF have the strongest effect on altering *ALPL* expression in the O-P1 sample. However, in the samples O-P2, O-P3, and Ch-P4, AA bFGF leads to a slight increase in *ALPL* gene expression. The treatment with DHA bFGF shows a similar effect; however, unlike AA bFGF, it also causes a decrease in expression in the O-P3 and Rm-P6 samples. When investigating the expression of *COL1A1*, the induction of its expression is most prominent in samples O-P3, Ch-P4, and L-P5 under AA bFGF treatment. Treatment with DHA bFGF maintained *COL1A1* expression, except for the O-P1 sample (Figure 4A).

### 3.4. GAPDH Protein Expression in Sarcoma CSCs Treated with AA and DHA

**Figure 5:**
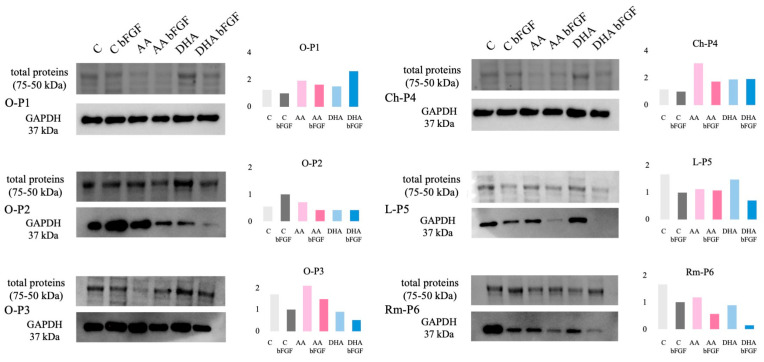
Analysis of the changes in GAPDH protein expression after the 7-day ascorbate treatment by Western blot. Total proteins were isolated following treatment of cancer stem cells derived from osteosarcoma samples, O-P1, O-P2, and O-P3, chondrosarcoma, Ch-P4, liposarcoma, L-P5, and rhabdomyosarcoma, Rm-P6, with ascorbic acid (AA), ascorbic acid combined with bFGF (AA bFGF), dehydroascorbic acid (DHA), and dehydroascorbic acid combined with bFGF (DHA bFGF). Total proteins visualized after transfer onto the nitrocellulose membrane (approximately range 75–50 kDa) are shown as a loading control for each sample. To account for the unequal protein loading, densitometric analysis was performed, and quantification of GAPDH protein bands was normalized to total protein lanes of each sample and presented as normalized data in the graphs.

Since the expression of GAPDH protein has proven to be very important when investigating the effect of ascorbate on CSCs, in this study, its expression was assessed by Western blot, with the total loaded protein shown as a control. While normalization to total protein loading is an accepted approach, we acknowledge that the addition of a dedicated loading control would further strengthen the reliability and accuracy of the data. As shown in Figure 5, on the blots, GAPDH showed quite uniform expression in samples O-P1, O-P3, and Ch-P4. However, the Ch-P4 sample treated with AA showed higher expression in the densitometric analysis. O-P3 shows a uniform expression of GAPDH on the blot, but the densitometric analysis shows that its expression rises in AA treatments and drops in DHA treatments. However, three of the analyzed samples, O-P2, L-P5, and Rm-P6, show downregulation in expression of GAPDH after 7-day treatment with AA and DHA on the blots, although it is somewhat less pronounced in the densitometric analysis. In the case of osteosarcoma CSC, O-P2, the highest expression is observed in the control with bFGF (C bFGF), while expression is reduced in all other treatments. The weakest bands are seen in the AA bFGF, DHA, and DHA bFGF treatments. In liposarcoma CSC, L-P5, it is observed that expression of GAPDH after the AA treatment is almost equal to C bFGF, while after the DHA treatment, expression is increased compared to the controls. However, after the AA bFGF and DHA bFGF treatments, GAPDH protein expression is considerably reduced. In the AA bFGF treatment, a very faint band is observed, and in the DHA bFGF treatment, no visible band is observed. In the case of rhabdomyosarcoma CSC, Rm-P6, there is nearly equal expression of GAPDH protein in the C bFGF control and the AA and DHA treatments. However, in the control without bFGF, GAPDH protein expression is greatly increased, as evidenced by a very intense band, while very faint bands are observed in the AA bFGF and DHA bFGF treatments, indicating a reduced expression of GAPDH protein when compared to the control C bFGF treatment (Figure 5).

### 3.5. Additional qPCR Analysis Reveals bFGF-Dependent Modulation of Oxidative Stress and Metabolic Genes in Sarcoma CSCs Treated with Ascorbate

Analysis of GAPDH protein expression in samples O-P2, L-P5, and Rm-P6 indicated its strong suppression at the protein level in treatments where AA and DHA were applied in the presence of bFGF. Therefore, to investigate how bFGF affects the impact of AA and DHA on sarcoma CSC, the expression of genes associated with oxidative stress and metabolism was analyzed again with additional analysis of samples treated without bFGF (Figure 4B). In sample O-P2, a stronger increase in CAT and SOD1 gene expression is observed in samples treated with DHA compared to AA, with the highest increase occurring in the DHA bFGF treatment. Regarding GPX1 gene expression, a uniform response cannot be observed across samples O-P2, L-P5, and Rm-P6. Accordingly, in O-P2 and Rm-P6, AA and DHA treatments cause a decrease in GPX1 gene expression compared to the control (C bFGF), while in sample L-P5, expression remained unchanged after treatment. On the other hand, AA and DHA treatments in the presence of bFGF in samples L-P5 and Rm-P6 show a consistent trend, leading to increased GPX1 expression compared to treatments without bFGF. Therefore, this effect is particularly evident in the differential expression of the *CAT* and *SOD1* genes, and to a somewhat lesser extent in the expression of the *GPX1* gene in L-P5 and Rm-P6. AA and DHA treatments in the presence of bFGF lead to increased expression of these enzymes compared to treatments without bFGF (Figure 4B).

Regarding the gene expression of *HIF1A*, in the sample O-P2, expression increases in the AA treatment without bFGF, while in all other treatments, expression levels remain almost the same as in the C bFGF control. In sample L-P5, *HIF1A* gene expression increases in all ascorbate treatments, with the increase being several times greater when bFGF is present in the treatment. In sample Rm-P6, *HIF1A* gene expression remains nearly unchanged in ascorbate treatments without bFGF, but in treatments where bFGF is present, expression increases approximately six-fold with AA bFGF and about seven-fold with DHA bFGF. Therefore, in the expression of *HIF1A*, a similar trend can be observed, as in the gene expression of genes related to oxidative stress (Figure 4B).

Regarding the expression of *GAPDH,* it was observed that its expression changes depending on the presence of bFGF in the culture medium. However, these changes were not as noticeable as at the protein level. Still, in the samples L-P5 and Rm-P6, *GAPDH* gene expression is higher in AA and DHA treatments when bFGF is present. However, in sample L-P5, AA bFGF and DHA bFGF treatments lead to a slight increase in *GAPDH* gene expression compared to the C bFGF control, whereas in sample Rm-P6, the addition of bFGF to ascorbate treatments (AA bFGF and DHA bFGF) maintains its expression similar to C bFGF. Generally, in treatments lacking bFGF (C, AA, and DHA), a decrease in *GAPDH* gene expression can be observed in all three samples compared to the C bFGF control (Figure 4B).

In the expression of the *PPARGC1A* gene, AA and DHA treatments had almost no effect on its expression in sample O-P2 compared to the control. In the samples L-P5 and Rm-P6, a strong increase in its expression was observed, which depends on the presence of bFGF in the culture medium during ascorbate treatments. Without bFGF, in AA and DHA treatments, the increase is much smaller or does not occur at all, with expression remaining at the C bFGF control level. AA bFGF and DHA bFGF treatments in sample Rm-P6 lead to a fivefold increase in *PPARGC1A* gene expression compared to the C bFGF control. In sample L-P5, this effect is even more pronounced, with DHA bFGF treatment resulting in a 16-fold increase and AA bFGF treatment in a 24-fold increase in expression compared to the C bFGF control (Figure 4B).

### 3.6. Mutation Analysis of Oncogenes and Tumor Suppressor Genes in Sarcoma CSCs Responsive to Ascorbate Treatment

Changes in the expression of genes related to oxidative stress, metabolism, as well as changes in GAPDH protein expression following AA and DHA treatments were observed in CSCs isolated from samples O-P2, L-P5, and Rm-P6. To assess whether some mutations in these samples could potentially contribute to the cytotoxic effects and influence the response of the cells to treatments, analysis of proto-oncogene and tumor suppressor genes was conducted, and the results are presented in Table 3.

It was found that sarcomas O-P2, L-P5, and Rm-P6 carry mutations in the *CTNNB1* (gene accession number: NG_013302.2), *KIT* (gene accession number: NG_007456.1), and *NF2* (gene accession number: NG_009057.1) genes. Sarcomas O-P2 and L-P5 have mutations in the *CTNNB1* gene, which encodes the β-catenin protein. In O-P2, a mutation was found at nucleotide 133 of the coding sequence, where thymine is replaced by cytosine (missense mutation). In L-P5, a mutation occurs at nucleotide 61, where guanine is replaced by adenine (missense gain-of-function mutation).

The *KIT* gene, which encodes the transmembrane receptor c-KIT, is a gene with the highest number of identified mutations: one in O-P2, one in Rm-P6, and four in L-P5. In O-P2, the mutation is at nucleotide 1676 of the coding sequence, where the switch of thymine to cytosine results in a change of valine to alanine. The same mutation was also detected in L-P5. Additionally, three more mutations were identified in L-P5, at nucleotide 1669, thymine is replaced by guanine, switching tryptophan to glycine. Furthermore, a deletion of 15 nucleotides after nucleotide 1673 was detected, resulting in the deletion of five amino acids. A fourth *KIT* mutation in L-P5 was identified at nucleotide 1727, where thymine is replaced by cytosine, changing leucine to proline. These mutations are located in the juxtamembrane region of the c-KIT receptor, a region that structurally still has not yet been fully resolved.

Mutations were also detected in the *NF2* gene, which encodes the Merlin protein. In L-P5, a mutation at position 655 of the coding sequence changes guanine to adenine, resulting in the substitution of valine with methionine at position 219 of the protein. In the same gene, a mutation was detected in Rm-P6 just a few nucleotides earlier—at position 624, cytosine is replaced by guanine, which results in the codon being read as a stop codon instead of glutamine, leading to a premature termination of Merlin protein synthesis.

## 4. Discussion

Although vitamin C has been investigated for cancer therapy for several decades now, its mechanisms of action in targeting cancer stem cells (CSCs) remain unclear. In this research, CSCs were isolated through a two-generation sphere-forming assay, which allowed the isolation of a more purified population of stem-like cells from an initially highly heterogeneous tumor mass. A major advantage of this study lies in the fact that the CSCs were isolated directly from sarcoma samples obtained from patients and not from established cell lines.

Following isolation through two generations of sphere assays, CSC characteristics were also confirmed by side population assay as well as by gene expression analysis of stem cell markers. Multiple scientific groups have already used the sphere assay to isolate the CSC population from sarcoma samples and cell lines. Palmini et al. confirmed osteosarcoma CSC stemness through sphere assay, surface marker expression (CD44, STRO-1, CD105), and RT-PCR gene expression of stemness-related genes (NANOG, POU5F1, SOX2) [26]. Next, Martins-Neves et al. showed that CSCs isolated from the MNNG/HOS cell line expressed higher levels of stemness markers and ABC transporters and formed significantly larger tumors in immunocompromised mice, confirming their tumorigenic potential [27]. Kim et al. found that CD133-positive cells from various sarcoma cell lines formed more spheres, suggesting a link between CD133 expression and CSC properties [28]. Similarly, Stratford et al. supported the CSC identity of CD133-positive, ALDH-active cells in liposarcoma using the sphere-forming method [29]. In this study, the side population assay was performed using a microtiter method developed by Seigel and Campbell [30]. This assay is usually conducted using a flow cytometer or FACS sorter, and, as demonstrated by Wu et al., Yang et al., and Yi et al., who investigated the side population in sarcoma, this assay confirmed the existence of side population cells in different sarcoma samples. Additionally, they concluded that side population cells had higher tumorigenic capacity when implanted into NOD/SCID mice [31,32,33].

As an additional method of characterization of CSC, gene expression analysis of ascorbate transporters GLUT1 (gene *SLC2A1*) and SVCT-2 (gene *SLC23A2*) was performed to assess whether there are differences in basal expression of these transporters at the level of mRNA. However, the expression of these genes between samples was quite similar, indicating there shouldn’t be differences in the uptake of ascorbate. This is important to take into account because, in hepatocellular carcinoma CSCs, Lv et al. showed that SVCT-2 gene expression is inversely proportional to the IC_50_ values of AA applied to the cells [34].

CSC derived from our sarcoma samples had created spheres through two generations, had dye efflux characteristic (except O-P1), as well as higher stemness marker gene expression compared to parental cell lines. Therefore, we conclude that a CSC-enriched population of cells was isolated from four different types of sarcomas. In addition to successful CSC isolation, their characteristics were also confirmed and found to be consistent with previously published data. Given that sarcomas are significantly less studied than carcinomas due to their lower incidence, these results are highly valuable as they confirm the presence of CSCs in four different types of sarcomas.

After the isolation and characterization of CSCs, the goal was to determine the optimal cytotoxic dose range for both ascorbic acid (AA) and dehydroascorbic acid (DHA). The concentrations of AA and DHA used in this study (0.05–10 mM) correspond to the pharmacologically achievable plasma levels following high-dose intravenous vitamin C administration, which can reach up to 20 mM in clinical trials [35]. Contrary to the AA, which is the most commonly used form due to its availability and stability, DHA has limited stability in solutions, which makes its clinical applicability more complex [36]. However, the advantage of DHA is the mechanism of its cellular uptake as it efficiently enters cells via GLUT transporters, which are highly expressed on tumor cells [37]. That is why, despite the pharmacokinetic and practical limitations of DHA, we wanted to investigate its effects in sarcoma CSCs, particularly because AA already has demonstrated cytotoxic activity against tumor cells, including cancer stem cells.

The experiments were performed using both established cell lines (as controls) and CSCs isolated from sarcomas. To account for the potential influence of bFGF, which is commonly used in culture media for CSC, we evaluated the cytotoxic effects of AA and DHA under both bFGF-supplemented and bFGF-deprived conditions. This approach allowed for the assessment of whether bFGF modulates the response of CSCs to the treatments with ascorbate. Firstly, cytotoxic tests performed on cell lines showed that, while AA had cytotoxic effects on all three cell lines (HEK293, U2OS, and hMSC), DHA did not show cytotoxic effects in cell lines HEK293 and hMSC, which suggests it is a good candidate for application in CSC. Secondly, tests on sarcoma CSC showed variability of cytotoxic effect of both AA and DHA, and also in regard to bFGF presence in the cultivation media. However, it is visible that DHA leads to a cytotoxic effect at lower concentrations than AA. Due to these results and the non-cytotoxic effect observed in control cell lines HEK 293 and hMSC, DHA has a good potential for selective targeting of sarcoma CSC. The concentration range at which both AA and DHA show their cytotoxic effect is in accordance with the results obtained previously by other groups. Chen et al. were among the first to investigate the cytotoxic effect of ascorbic acid (AA) in vitro, and they investigated the effect of pharmacological doses of ascorbate on 10 different tumor cell lines as well as on four normal cell lines. Their results are highly important since they showed how ascorbate can have a selective cytotoxic effect on tumor cells at pharmacological doses. The IC_50_ values for ascorbic acid ranged between 1 and 20 mM, depending on the tumor type [38].

Additionally, Valenti et al. reported a significant decrease in the viability of MG-63 human osteosarcoma cells after 24 h treatment with 1 mM ascorbic acid (AA) [39]. In a more recent study, Vaishampayan et al. investigated the cytotoxic effects of AA, AA-2-phosphate, and DHA on several osteosarcoma cell lines, including U2OS. AA showed the strongest effect, with IC_50_ for U2OS at 5.4 mM, while DHA had a much weaker effect, with no IC_50_ reached even at 20 mM [40]. A study by Jovičić et al., previously published by our group, demonstrated that osteosarcoma CSCs exhibited the highest sensitivity to ascorbic acid treatment, with an IC_50_ of 15.5 mM, compared to control and parental cell lines [41]. The effect of ascorbic acid was also explored in hepatocellular carcinoma as well as breast cancer. Various studies have shown that achieving a selective effect requires millimolar doses of ascorbate. For example, in the study about hepatocellular carcinoma, it was found that in Huh7 and Hep3B CSCs, cytotoxic effect was achieved between 0.5 mM and 1 mM dose [42]. In studying breast cancer, it was found that the MCF-7 cell line has a population of cells rich in mitochondria, with a high capacity to form mammospheres, which could be inhibited by a 1 to 2 mM dose of ascorbate [43]. In a study by Lv et al., a correlation was found between expression of vitamin C transporters and cell sensitivity. However, in our study, we did not find such a connection, possibly because we followed gene rather than protein expression of the transporters [34]. Based on the previously mentioned findings, as well as the results obtained in this study, pharmacologic concentrations of ascorbate are required to exhibit a cytotoxic effect in CSCs from sarcomas, as well as in CSCs from other cancer types.

It is important to point out that the difference in the effects of the two forms of ascorbate lies in their structure and the mechanisms by which they enter cells. When ascorbate enters the cells as DHA via the GLUT transporters, it is rapidly reduced back to AA through the action of glutathione and other cellular enzymes, leading to the depletion of intracellular antioxidant reservoirs that are essential for maintaining redox balance [12,16]. However, it is possible that providing cells with a higher initial concentration of DHA instead of AA could result in a greater exhaustion of antioxidant defense mechanisms, since AA uptake does not require this reduction step that occurs when large amounts of DHA enter the cells through the GLUTs [13]. Future studies should consider including a broader range of ascorbate concentrations between 1 mM and 10 mM. This would provide more precise insight into the IC_50_ values for sarcoma CSCs treated with both AA and DHA and help to better understand the dose-dependent effects of these compounds.

After establishing the range of concentrations that can be applied to sarcoma CSCs, we wanted to investigate the underlying mechanisms of the cytotoxic effect of ascorbate in sarcoma CSCs during the early stages of cellular response to the treatment. Since the goal was to observe the effect of treatments after 7 days, and not the instant effects of the treatments, changes in gene expression were investigated. Given that cytotoxic effects in most CSCs isolated from sarcoma samples were observed at ascorbate concentrations between 1 and 5 mM, a dose of 1 mM was selected for application over a 7-day period. This way, we were able to detect changes in CSC metabolism and oxidative stress defense before ascorbate causes the cytotoxic effect.

Previous studies have demonstrated that vitamin C, when applied at concentrations above 1 mM, induces oxidative stress in CSCs as well as in other cancer cells [34,38,40,44]. In this study, increased expression of genes for enzymes that regulate oxidative stress in osteosarcoma O-P2 and liposarcoma CSCs indicates that these cells were exposed to oxidative stress. In contrast, other CSC samples showed minimal changes in gene expression, suggesting either the absence of oxidative stress or efficient cellular mechanisms for its removal. When investigating the effects of vitamin C in osteosarcoma cell lines, Vaishampayan et al. found that treatment with AA resulted in significantly higher levels of ROS than DHA at concentrations above 5 mM, leading to cell death within 24 h [40]. This pro-oxidative effect is associated with the presence of intracellular iron and the Fenton reaction, which increases both intra- and extracellular hydrogen peroxide (H_2_O_2_). However, it is important to note that Vaishampayan et al. found that when ascorbate was applied in the 0.5 mM–20 mM range, they did not observe the same effect for DHA. Generally, several studies have shown that oxidative stress induced by AA can lead to cytotoxicity in CSC-like cells, including hepatocellular carcinoma and neural stem cells, especially when antioxidant systems such as glutathione (GSH) are depleted [34,45,46]. This effect can also be linked to the connection between ROS and GAPDH depletion, first shown by Yun et al. in 2015 in a study on *KRAS* and *BRAF* mutant colorectal cancer cells [18]. Their study was highly significant and laid the foundation for future research on the relationship between oxidative stress and GAPDH after the vitamin C treatment in cancer cells.

To examine gene expression of genes connected with changes in metabolism, we investigated gene expression of *GAPDH* and *PPARGC1A*. Although *GAPDH* mRNA expression remained largely unchanged, Western blot analysis at the protein level revealed suppression of GAPDH in osteosarcoma (O-P2), liposarcoma (L-P5), and rhabdomyosarcoma (Rm-P6) CSC samples. This effect was especially evident in ascorbate treatments administered in the presence of bFGF, where on the blots, GAPDH protein was barely detectable. A limitation of these results is that Western blot data were derived from a single experiment, and further biological replicates would be required to confirm the observed trends. This effect was already noticed, and GAPDH silencing was connected to S-glutathionylation [18]. However, what intrigued us was that this effect was enhanced by the presence of bFGF in the cell culture media. Expression analysis of *PPARGC1A*, encoding the mitochondrial biogenesis regulator PGC-1α and indicative of a metabolic shift toward oxidative phosphorylation, demonstrated upregulation in cancer stem cells derived from liposarcoma and rhabdomyosarcoma [47,48]. In addition to being a transcription regulator of cellular metabolism, it also participates in cellular detoxification from ROS, as it induces expression of superoxide dismutase 2, catalase, and glutathione peroxidase [49]. These data indicate that CSC of L-P5 and Rm-P6 activate mechanisms for fighting oxidative stress, which is happening due to extensive import of both AA and DHA. Furthermore, regarding *HIF1A* expression, we observed increased gene expression after 7-day treatments, particularly in the presence of bFGF. Although vitamin C is generally known to promote HIF-1α degradation via prolyl hydroxylases under normoxia [13], we observed an upregulation of *HIF1A* mRNA following both AA and DHA treatment. Partly this effect can be attributed to the presence of bFGF in the cultivation media, which is known to enhance HIF-1α expression and activity through the PI3K/AKT and MAPK/ERK signaling pathways [50]. Also, oxidative stress is known to stabilize HIF-1, a key factor in metabolic reprogramming. Its activation promotes anaerobic glycolysis while suppressing oxidative phosphorylation by upregulating GLUT transporters and glycolytic enzymes such as glucokinase, aldolase A, and LDH-M. Consequently, HIF-1 redirects metabolism toward glycolysis, in part by enhancing glucose uptake through GLUT proteins [9,13].

To assess the potential differentiation effect of AA and DHA, gene expression of *ALPL* and *COL1A1* was analyzed. AA mainly increased *ALPL* levels, unlike DHA, especially in osteosarcoma and chondrosarcoma CSCs. Upregulation of *COL1A1* was more pronounced in chondrosarcoma and liposarcoma CSCs, and less in osteosarcoma. However, it is important to note that achieving a differentiation effect is challenging with bFGF supplementation in the culture medium, as bFGF promotes stemness and maintains the differentiation potential of CSCs. Previously, Valenti et al. demonstrated that AA concentrations up to 1 mM stimulated differentiation in the MG-63 human osteosarcoma cell line—evidenced by increased osteocalcin and ALP expression—while 1 mM AA reduced RUNX2 expression, a transcription factor linked to osteoblast differentiation. Also, the authors emphasized that AA can have different effects on MG-63 cells: at lower concentrations, it promoted differentiation, while at 1 mM, it induced apoptosis [39]. Regarding our results, we can conclude that during a 7-day treatment, AA did start to induce differentiation in some samples, but this process cannot be connected to the cytotoxic effect.

Given that mutations in critical regulatory proteins may alter pathways involved in oxidative stress, metabolism, and cellular differentiation, we investigated the presence of oncogenic and tumor suppressor mutations in our sarcoma CSC samples to assess their potential contribution to ascorbate treatment response. In this study, we used the Qiagen qBiomarker Somatic Mutation PCR Array to identify the most frequent and biologically relevant mutations in soft tissue sarcomas. However, this type of targeted approach provides only a limited view of the mutational landscape, and future studies employing comprehensive sequencing should explore these mechanisms in greater detail. Regarding our results, in osteosarcoma O-P2 and liposarcoma L-P5, mutations were found in the N-terminal domain of β-catenin, which can lead to stabilization of β-catenin and its nuclear accumulation, even in the absence of Wnt signaling. This results in constitutive activation of self-renewal genes, supporting CSC maintenance and resistance [33]. A study on pancreatic cancer highlighted a link between vitamin C and the Wnt/β-catenin signaling pathway. High-dose vitamin C treatment (4–5 mM) downregulated the levels of non-phosphorylated β-catenin, which suppressed Wnt/β-catenin signaling and had anti-tumor effects [51]. Additionally, the mutations in β-catenin are especially interesting because of the activation of Wnt signaling, particularly through stabilization of β-catenin, which is connected to elevated expression of GLUT1 and enables survival of cancer cells [52]. In the cases of exposure to DHA, these kinds of mutations could make the cells more sensitive to the DHA treatment.

Next, mutations in the gene for c-KIT, a transmembrane receptor tyrosine kinase, were detected in all three patients. These mutations enable the activation of the receptor even in the absence of ligand [53,54]. Activated c-KIT regulates proliferation, survival, and migration, primarily through downstream signaling cascades. Phosphorylation at tyrosine 721 activates the PI3K/AKT pathway, promoting survival, while phosphorylation at tyrosine 730 activates PLC-γ signaling, contributing to proliferation and evading cell death [53,55]. The *NF2* gene encodes the tumor suppressor protein Merlin [56], which consists of three different domains that enable the protein to adopt either an inactive open or active closed conformation [56,57]. A nonsense mutation at amino acid 212 produces a truncated protein that impairs Merlin’s tumor suppressor role. The V219M missense mutation, located in the region critical for conformational switching, may stabilize its inactive state, though this remains poorly described in the literature [57]. Active Merlin inhibits several oncogenic pathways, and its loss leads to increased proliferation, enhanced survival, and resistance to apoptosis [57,58]. Collectively, the detected mutations contribute to enhanced cell survival, proliferation, and evasion of regulatory mechanisms, thereby shifting cellular signaling pathways toward resistance to cell death. These properties make cancer stem cells (CSCs) more resistant and, therefore, more difficult to target with anti-tumor therapies.

Considering all these results, there is an indication for interaction between the effects of DHA and proliferative signals, which are influenced both by bFGF supplementation in the culture medium and by mutations present in the rhabdomyosarcoma and liposarcoma samples, ultimately contributing to the observed cytotoxic effect. Although this effect is quite counterintuitive, some publications are suggesting the approach of rather turning on proliferative signals in cancer cells to the point that they “burn out” [59,60]. Similarly, sustained proliferative signals from bFGF and mutations in these samples can, over time, lead to exhaustion of regulatory mechanisms for managing oxidative stress in CSCs. This effect is enhanced by prior depletion of these defense mechanisms due to continuous uptake of DHA through glucose transporters. Therefore, this could be a new approach towards targeting sarcoma CSCs and inspire new ideas for “paradoxal intervention” suggested by Dias and Bernards [59].

## 5. Conclusions

This study’s investigation into sarcoma stem cell sensitivity to vitamin C demonstrates that DHA treatment can lead to a desired cytotoxic effect in CSCs. Since self-renewal signals driven by bFGF supplementation and oncogenic mutations influence the cytotoxicity of DHA, it is essential to determine the point at which these signals transition from promoting cell survival and proliferation to inducing cell death. Understanding how this shift relates to DHA treatment is crucial for optimizing its cytotoxic effect. Our findings highlight that DHA selectively targets sarcoma CSCs at lower concentrations than AA, with metabolic shifts and oxidative stress responses playing key roles in this process. These insights offer new perspectives for targeting CSCs by exploiting their metabolic vulnerabilities and suggest potential for “paradoxical interventions” that harness proliferative exhaustion. Overall, this work provides valuable knowledge for developing more effective therapies against sarcoma through selective CSC targeting.

## Figures and Tables

**Table 1:** Primer sequences for gene expression analysis of stemness markers.

Gene	Gene Accession Number	Primer	Primer Sequence
*ACT*	NM_001101.5	upstream	CACCATTGGCAATGAGCGGTTC
		downstream	AGGTCTTTGCGGATGTCCACG
*SLC2A1*	NM_006516	upstream	TTGCAGGCTTCTCCAACTGGAC
		downstream	CAGAACCAGGAGCACAGTGAAG
*SLC23A2*	NM_203327.2	upstream	CCAAGAAAGGATGGACTGCGTAC
		downstream	CATCTGTGCGAGCATAGAAGCC
*NANOG*	NM_024865	upstream	CTCCAACATCCTGAACCTCAGC
		downstream	CGTCACACCATTGCTATTCTTCG
*POU5F1*	NM_002701	upstream	CCTGAAGCAGAAGAGGATCACC
		downstream	AAAGCGGCAGATGGTCGTTTGG
*NES*	NM_006617.2	upstream	TCAAGATGTCCCTCAGCCTGGA
		downstream	AAGCTGAGGGAAGTCTTGGAGC

**Table 2:** Primer sequences for gene expression analysis of markers of oxidative stess, metabolism and differentiation.

Gene	Gene Accession Number	Primer	Primer Sequence
*ACT*	NM_001101.5	upstream	CACCATTGGCAATGAGCGGTTC
		downstream	AGGTCTTTGCGGATGTCCACG
*SOD1*	NM_000454.5	upstream	CTCACTCTCAGGAGACCATTGC
		downstream	CCACAAGCCAAACGACTTCCAG
*CAT*	NM_001752.4	upstream	GTGCGGAGATTCAACACTGCCA
		downstream	CGGCAATGTTCTCACACAGACG
*GPX1*	NM_001329502.2	upstream	GCGGGGCAAGGTACTACTTA
		downstream	CTCTTCGTTCTTGGCGTTCT
*HIF1A*	NM_001530	upstream	TATGAGCCAGAAGAACTTTTAGGC
		downstream	CACCTCTTTTGGCAAGCATCCTG
*GAPDH*	NM_002046.7	upstream	TCAAGGCTGAGAACGGGAAG
		downstream	CGCCCCACTTGATTTTGGAG
*PPARGC1A*	NM_013261	upstream	CCAAAGGATGCGCTCTCGTTCA
		downstream	CGGTGTCTGTAGTGGCTTGACT
*ALPL*	NM_000478	upstream	GCTGTAAGGACATCGCCTACCA
		downstream	CCTGGCTTTCTCGTCACTCTCA
*COL1A1*	NM_000088	upstream	GATTCCCTGGACCTAAAGGTGC
		downstream	AGCCTCTCCATCTTTGCCAGCA

**Table 3:** Somatic mutations discovered in selected sarcoma samples.

*Gene*	*COSMIC ID*	*nt Change*	*AA Change*	*O-P2*	*L-P5*	*Rm-P6*
*CTNNB1*	5663	c.133T>C	p.S45P	Mutant	WT	WT
*CTNNB1*	5738	c.61G>A	p.A21T	WT	Mutant	WT
*CTNNB1*	5661	c.94G>T	p.D32Y	WT	WT	WT
*KIT*	28026	c.1621A>C	p.M541L	WT	WT	Mutant
*KIT*	1221	c.1669T>G	p.W557G	WT	Mutant	WT
*KIT*	18896	c.1673_168del15	p.K558_E562del	WT	Mutant	WT
*KIT*	1255	c.1676T>C	p.V559A	Mutant	Mutant	WT
*KIT*	1290	c.1727T>C	p.L576P	WT	Mutant	WT
*NF2*	22240	c.634C>T	p.Q212 *	WT	WT	Mutant
*NF2*	22454	c.655G>A	p.V219M	WT	Mutant	WT
*NF2*	22000	c.784C>T	p.R262 *	WT	WT	WT
*TP53*	10758	c.659A>G	p.Y220C	WT	WT	WT

* represents substitution of the original amino acid with a stop codon, leading to protein truncation.

## Data Availability

The original contributions presented in this study are included in the article. Further inquiries can be directed to the corresponding author.
